# Expansion of Rare and Harmful Lineages is Associated with Established Rheumatoid Arthritis

**DOI:** 10.3390/jcm9041044

**Published:** 2020-04-07

**Authors:** Natalia Mena-Vázquez, Patricia Ruiz-Limón, Isabel Moreno-Indias, Sara Manrique-Arija, Francisco J. Tinahones, Antonio Fernández-Nebro

**Affiliations:** 1 The Institute of Biomedical Research in Malaga (IBIMA), 29010 Málaga, Spain; nataliamenavazquez@gmail.com (N.M.-V.); patrilimon@hotmail.com (P.R.-L.); sarama_82@hotmail.com (S.M.-A.); fjtinahones@uma.es (F.J.T.); afnebro@gmail.com (A.F.-N.); 2 UGC Rheumatology, Regional University Hospital of Malaga, University of Malaga, 29009 Málaga, Spain; 3 Clinical Management Unit of Endocrinology and Nutrition, Hospital Universitario Virgen de la Victoria, 29010 Málaga, Spain; 4 CIBER Physiopathology of Obesity and Nutrition (CIBEROBN), Carlos III Health Institute, 28029 Madrid, Spain; 5 Department of Medicine, University of Malaga, 29010 Málaga, Spain

**Keywords:** rheumatoid arthritis, gut microbiota, anti-citrullinated protein antibodies, *Collinsella aerofaciens*

## Abstract

Objectives: To characterize the gut microbiota profile in rheumatoid arthritis (RA) patients and investigate its association with certain characteristics of RA. Patients and methods: A nested case–control cohort of 40 patients with RA and 40 sex-age matched controls was studied. Subjects with diabetes, with any other inflammatory disease, practicing extreme diets, taking antibiotics, probiotics or under any new treatment for at least three months prior to sampling were excluded. The microbiota composition was determined by 16S rRNA pyrosequencing and bioinformatics analysis by Quantitative Insights Into Microbial Ecology (QIIME). Other variables included clinical-laboratory variables and average Disease Activity Score 28 points during the follow-up period. Multiple linear regression models were constructed to investigate the possible risk factors for the microbiota. Results: β-diversity data showed that patients tend to differ from healthy subjects according to their microbiota (*p* = 0.07). The analysis showed an increase in *Collinsella aerofaciens*, *Sedimentibacter* and *Enterococcus* genera in patients compared to controls, as well as a decrease in *Dorea formicigenerans*. Likewise, an increase in the activity of arginine deiminase was observed, which was found in approximately 90% of the RA genes of the genus *Collinsela*. The sequence number of *Collinsella aerofaciens* was independently associated with age (B (95%CI), −0.347 (−21.6, −2.1)), high ACPA (0.323 (27.4–390.0)) and smoking (0.300 (8.8–256.4)) in RA patients. In addition, we observed decreases in *Sarcina*, *02d06* and *Porphyromonas* bacterial lineages. Conclusion: Patients with RA present dysbiosis, resulting from an abundance of certain bacterial lineages and a decrease in others. These alterations could influence the maintenance of autoimmunity to this disease.

## 1. Introduction

Rheumatoid arthritis (RA) is a systemic autoimmune disorder that causes joint swelling, deformity, and dysfunction. Most patients with RA produce autoantibodies (rheumatoid factor (RF) and anti-citrullinated protein antibody (ACPA)), which are associated with risk of developing RA and can predict severe disease [1].

It is thought that RA results from an interaction between genetic, environmental, hormonal, and immunopathological factors [2]. Data from recent studies suggest that RA begins to develop after exposure of the mucous membranes to environmental factors [3]. Diet and intestinal microbiota can modify intestinal barrier strength, functional integrity, and regulation of permeability [4].

Studies on human intestinal microbiota and RA suggest that affected patients have different degrees of dysbiosis and poorer microbial diversity than controls [5,6,7,8,9,10,11,12,13,14,15]. The findings also suggest that chronic inflammation of the gut is characterized by a shift from a symbiotic to a dysbiotic community. Dysbiosis may cause a local imbalance between tolerance and immunity, which may spread to other distant tissues. This imbalance may occur through mechanisms such as: ATP-stimulated Th17 cells activated by commensal bacteria [16], molecular mimicry [14,17], citrullination of proteins [18], or translocations of bacteria from the mucous membrane to joints [19]. Citrullination of bacterial and human proteins can expose hidden epitopes, leading to loss of tolerance and to the production of ACPA [20].

The aim of the present study was to characterize the gut microbiota profile and investigate whether there is an association between gut dysbiosis, inflammatory activity, and prognostic factors in patients with established RA.

## 2. Patients and Methods

### 2.1. Study Population

We performed a cross-sectional study of 40 patients with RA and 40 sex- and age-matched healthy controls from the same geographical area. Patients (aged ≥16 years) with RA were selected from a cohort of incident cases of RA recruited between 2007 and 2011 and followed prospectively until today. RA patients were classified according to the 2010 criteria of the American College of Rheumatology/European League against Rheumatism [21]. The exclusion criteria were presence of inflammatory or rheumatic diseases other than RA (except for secondary Sjögren’s syndrome), diabetes, or any non-controlled general condition. We also excluded patients and controls with extreme diets, those exposed to antibiotic therapy (current or previous three months), those taking probiotic agents, and those who had started a new treatment.

All subjects gave their informed consent for inclusion before they participated in the study. The study was conducted in accordance with the Declaration of Helsinki, and the protocol was approved by the Ethics Committee of Málaga (“Comité de Ética de la Investigación de Málaga”). (Project identification code 4/2016, P19).

### 2.2. Clinical and Laboratory Variables

Patients were assessed using a standardized clinical interview and clinical analysis before enrollment. Demographic, clinical, laboratory, and treatment-related data were recorded by a rheumatologist. The Disease Activity Score-28 with Erythrocyte Sedimentation Rate (DAS28-ESR) [22] and health assessment questionnaire (HAQ) [23] were estimated at baseline and during follow-up. Moderate-to-high activity was defined as a DAS28-ESR score of ≥3.2. The mean DAS28-ESR and HAQ values were used as summary variables during follow-up.

### 2.3. Sample Collection and DNA Extraction

Peripheral venous blood samples were collected after eight hours of fasting. Fecal samples were refrigerated immediately and transported to the laboratory, where they were stored at −80 °C for subsequent analysis. DNA was extracted from 200 mg of stool samples using the QIAamp DNA stool Mini kit (Qiagen, Hilden, Germany) following the manufacturer’s recommendations. DNA concentration and purity were determined with a Nanodrop spectrophotometer (Nanodrop Technologies, Wilmington, DE, USA).

### 2.4. 16. S Sequencing

Ribosomal 16S rRNA gene sequences were amplified using the 16S Metagenomics Kit (Thermo Fisher Scientific Inc., Waltham, MA, USA), consisting of primer pools to amplify multiple variable regions (V2-4–8 and V3–6) [7,8,9] of the 16S rRNA. The libraries were created using the Ion Plus Fragment Library Kit (Thermo Fisher Scientific Inc., Waltham, MA, USA). Barcodes were added to each sample using the Ion Xpress Barcode Adapters kit (Thermo Fisher Scientific Inc., Waltham, MA, USA). Emulsion PCR and sequencing of the amplicon libraries were performed on an Ion 520 chip (Ion 520^TM^ Chip Kit) via the Ion Chef System and Torrent S5^TM^ system, respectively, using the Ion 520^TM^/530^TM^ Kit-Chef (Thermo Fisher Scientific Inc., Waltham, MA, USA). Base calling and run demultiplexing were performed using Torrent Suite^TM^ Server software (Thermo Fisher Inc., Waltham, MA, USA), version 5.4.0, with default parameters for 16S Target Sequencing (bead loading ≤ 30, key signal ≤ 30, and usable sequences ≤ 30).

### 2.5. Bioinformatic Processing

The open source Quantitative Insights Into Microbial Ecology (QIIME) 1.9.1 was used to analyze sequence quality, as previously described by our group [24,25]. The representative sequences were processed using the UCLUST algorithm to assign the taxonomy, and the relative abundance of each operational taxonomic unit (OTU) was determined using the Greengenes 16S rRNA gene database. A random sub-sample with the same number of sequences was used to evaluate alpha and beta diversity through QIIME, and the OTUs were aligned with PyNAST in order to build a phylogenetic tree.

### 2.6. Phylogenetic Investigation of Communities by Reconstruction of Unobserved States (PICRUSt) Analysis

PICRUSt analysis was used to predict metagenome function by picking OTUs from the Greengenes database, as described elsewhere [26]. The resulting OTU table was used to predict the metabolic pathways of the metagenome at three different levels in the Kyoto Encyclopedia of Genes and Genomes (KEGG) Orthology (KO) (Level (L) 1 to L3). Arginine deiminase gene content was predicted using the metagenome_contributions.py script from the KEGG orthology K01478 [EC:3.5.3.6], arginine deiminase.

### 2.7. Statistical Analysis

The open source Statistical Analysis of Metagenomic Profiles (STAMP (v 2.1.3)) [27] was used to compare the abundance of taxa and KEGG categories and subcategories between RA patients and controls. α diversity was assessed based on a nonparametric Student *t* test with 999 Monte Carlo permutations (default number); ß diversity was assessed based on an analysis of similarities (ANOSIM) with 99 permutations. *p*-values were corrected for multiple comparisons using the Benjamini-Hochberg method when appropriate.

Data are presented as mean (SD), median (IQR), or totals with percentages. Normality was tested using the Kolmogorov-Smirnov Test. The baseline characteristics were compared between groups using the χ^2^ test, a two-tailed *t* test (Fisher’s exact test when necessary), or the Mann-Whitney test.

The associations between microbiome (dependent variable) and risk factors were studied using binomial logistic regression models. Statistical analyses were performed with IBM SPSS Statistics 25 (IBM, Armonk, NY, USA).

## 3. Results

Table 1 shows the characteristics of patients and healthy controls. Although there were no differences between the groups for most epidemiological parameters and comorbidities, a higher percentage of RA patients were former smokers. Most patients had positive RF and ACPA titers and low disease activity, and all patients were under treatment with disease-modifying anti-rheumatic drugs (DMARDs), mainly methotrexate followed by biologic therapy.

### 3.1. Analysis of the Diversity and Similarity of Gut Microbiota in RA Patients and Controls

**Figure 1:**
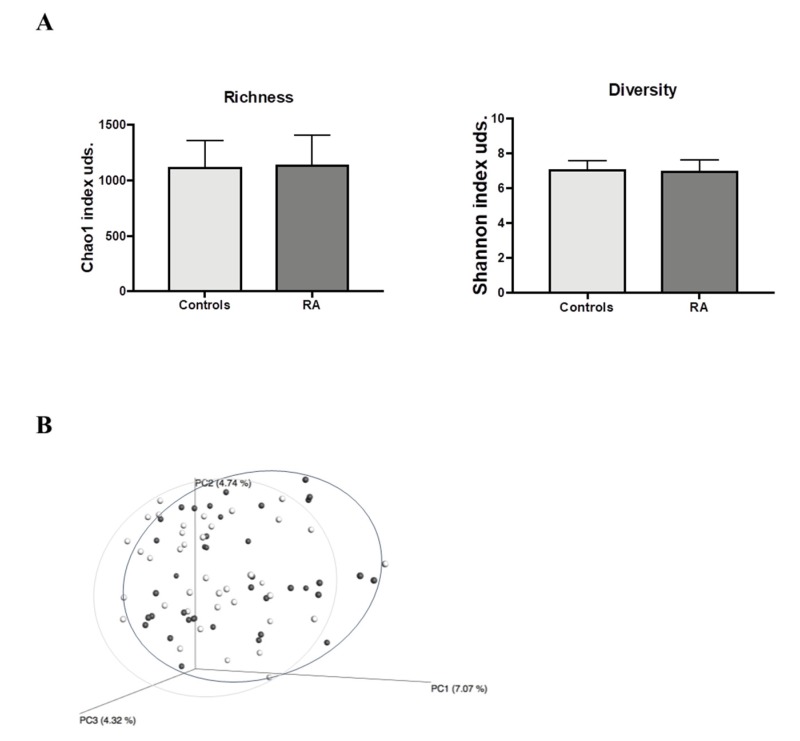
Fecal bacterial community structure in RA patients and controls (**A**) Richness (Chao-1 Index) and diversity (Shannon Index) of RA patients and controls. (**B**) Principal coordinate analysis (PCoA) using unweighted UniFrac distances. Each point corresponds to a community coded according to the group. The percentage of variation explained by the plotted principal coordinates is indicated on the axes. RA patients (dark grey dots), controls (light grey dots). Analysis of similarities (ANOSIM) *p* = 0.070. RA, Rheumatoid arthritis.

Once the quality assessment was complete, a total of 3,700,204 quality 16S rRNA gene sequences, with an average of 47,438.5 sequences per sample, had passed through the filters, which were applied by means of QIIME. The microbiota of all fecal samples comprised 14,304 OTUs with a 97% similarity cut-off. Our analysis showed that α-diversity (Shannon index), and richness (Chao 1 index) did not result in any significant differences in the gut microbiota profiles between RA patients or controls (Figure 1A).

ß-diversity was calculated by unweighted UniFrac distance and showed that RA patients tended to differ from controls (ANOSIM test, *p* = 0.07). Both populations were clustered according to principal coordinate analysis (PCoA) (Figure 1B).

### 3.2. Phylogenetic Differences in Gut Microbiota Between RA Patients and Controls

**Figure 2:**
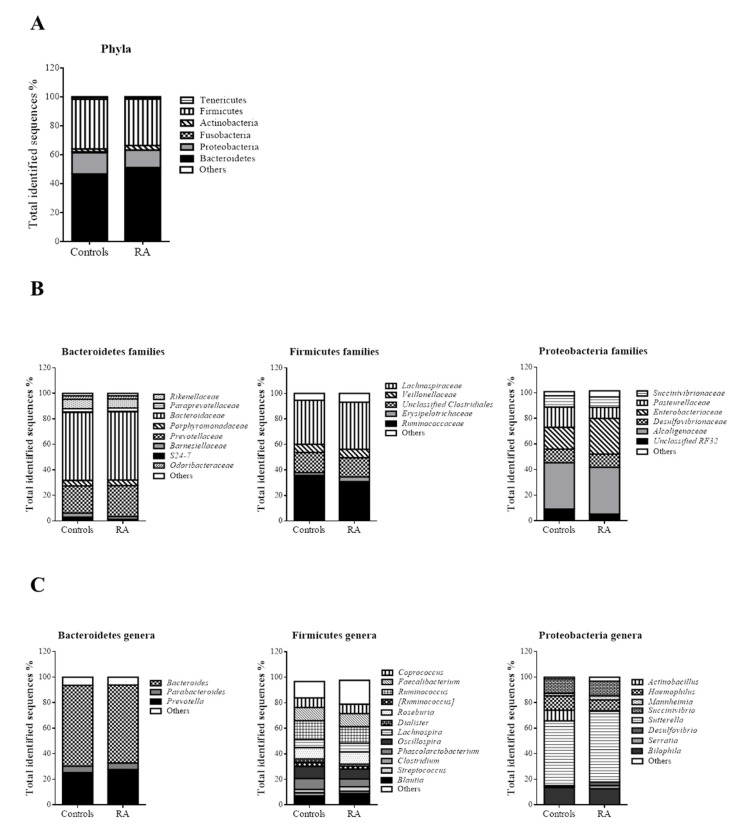
(**A**) Phylogeny at phyla in controls and RA patients. (**B**) Microbial community structure at the family level in fecal samples of controls and RA patients (Bacteroidetes families, Firmicutes families and Proteobacteria families). (**C**) Relative abundance of predominant genera in the microbiota of controls and RA patients (Bacteroidetes genera, Firmicutes genera and Proteobacteria genera). Data are shown as a percentage of the total identified sequences per group.

The most abundant OTUs were found within those belonging to the Bacteroidetes phylum (46.60% controls vs. 51.03% RA patients, *p* = 0.096), Firmicutes phylum (34.50% controls vs. 32.13 RA patients, *p* = 0.310), and Proteobacteria phylum (14.80% controls vs. 12.10% RA patients, *p* = 0.234) (Figure 2A).

At the family level (Figure 2B), significantly higher values were observed in RA patients for *Enterococcaceae* (0.05% RA patients vs. 0.01% controls; *p* = 0.009), *Comamonadaceae* (0.168% RA patients vs. 0.061% controls; *p* = 0.027), *Moraxellaceae* (0.061% RA patients vs. 0.016% controls; *p* = 0.038), and *Eubacteriaceae* (0.01% RA patients vs. 0% controls; *p* = 0.05).

Significant differences between the groups were also found at the genus level. The genera *Enterococcus* (*p* = 0.008), *Sedimentibacter* (*p* = 0.037), and *Collinsella* (*p* = 0.037) were significantly more frequent in RA than in controls. Conversely, the genera *Sarcina* (*p* = 0.013), *02d06* (*p* = 0.023), and *Porphyromonas* (*p* = 0.031) were significantly less frequent in RA patients than in controls (Figure 2C).

**Figure 3:**
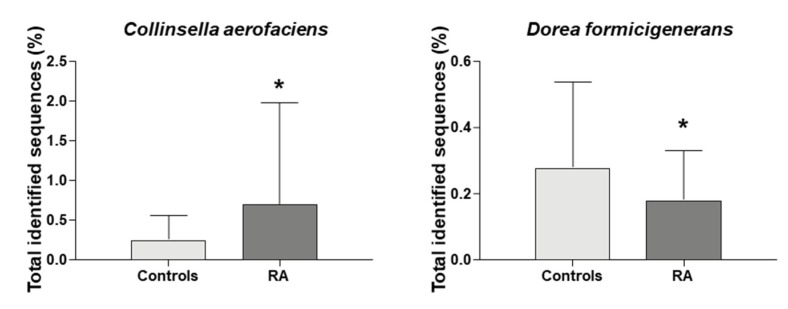
Microbial composition at species level of *Collinsella aerofaciens* and *Dorea formicigenerans* * indicates significant differences vs. controls (*p*-value ≤ 0.05). RA, Rheumatoid arthritis.

Interestingly, at the species level we identified a significant increase in the abundance of *Collinsella aerofaciens* (*p* = 0.039) and a significant decrease in *Dorea formicigenerans* (*p* = 0.044) in RA patients than in controls (Figure 3).

### 3.3. Differences in the Metabolic Profiles of Gut Microbiota Between RA Patients and Controls

**Figure 4:**
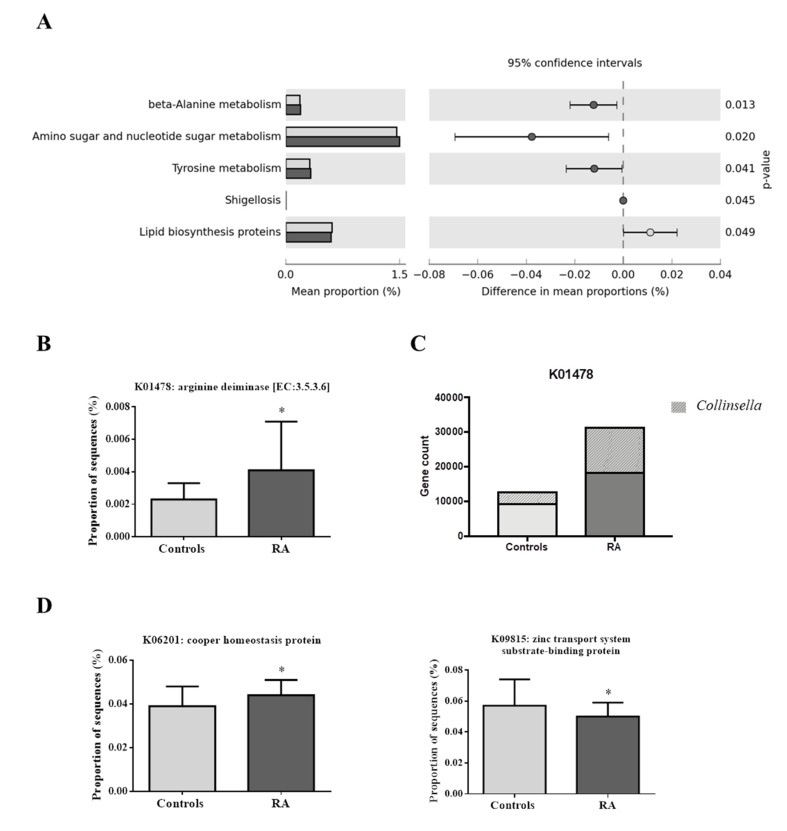
(**A**) Significant differences in metabolic capacities of the gut microbiota among controls (light grey) and RA patients (dark grey). Only functional capacities with *p*-value < 0.05 are shown. (**B**) Kyoto Encyclopedia of Genes and Genomes (KEGG) category showed significant differences in the enzyme arginine deiminase, between controls and RA patients. (**C**) Arginine deiminase gene count in controls and RA patients, and *Collinsella* contribution. (**D**) KEGG category showed significant differences in the copper homeostasis protein and zinc transport system substrate-binding protein among controls and RA patients. * indicates significant differences vs. controls (*p*-value < 0.05). RA, Rheumatoid arthritis.

Our analysis revealed no significant differences between the groups in the predicted functions of the highest levels 1 and 2 of the KEGG orthology. However, in level 3 of the KO categories, significant functional annotations in the microbiota of RA patients were observed for “beta alanine metabolism” (*p* = 0.013), “amino sugar and nucleotide sugar metabolism” (*p* = 0.020), “tyrosine metabolism” (*p* = 0.041), and “Shigellosis” (*p* = 0.045). “Lipid biosynthesis proteins” were significantly more frequent in controls than in RA patients (*p* = 0.049) (Figure 4A).

In a further analysis with Phylogenetic Investigation of Communities by Reconstruction of Unobserved States (PICRUSt) results, we focused on the enzyme arginine deiminase. Higher levels of this gene were found on RA patients (*p* = 0.0041) (Figure 4B). Our results show that the levels of the arginine deiminase gene were higher in RA patients (27985.97 copies) than in controls (16361.64 copies) (Figure 4C). Finally, in order to recognize the taxa contributing to the arginine deiminase gene count, further analysis revealed that the genus *Collinsella,* and mainly its species *C. aerofaciens,* highly contributed to the difference in gene counts between RA patients (33.71%) and controls (20.75%) (Figure 4C).

Moreover, we found copper homeostasis protein to be significantly more frequent (*p* = 0.017) and zinc transport system substrate binding-protein to be significantly less frequent (*p* = 0.039) in RA patients than in controls (Figure 4D).

### 3.4. Associations Between Clinical Characteristics of RA Patients and Collinsella aerofaciens

There was a greater representation of the number of *C. aerofaciens* sequences in RA patients with high ACPA titers, patients taking biologic therapy, and current or former smokers. However, no differences were observed between the number of *C. aerofaciens* sequences and the activity score or other DMARDs (Table 2).

In the multivariate analysis (Table 3), the sequence number of *C. aerofaciens* was independently associated with age, high ACPA titers, and a history of smoking in RA patients.

## 4. Discussion

Most studies of dysbiosis in RA report lower microbial diversity characterized by the expansion of some microbial lineages, along with the contraction of others [14,28,29]. Compared with controls, the RA patients in our study displayed lower β and similar α diversity. Although our data yielded no more than a statistical trend, the patients included in our study responded well to treatment, which could have restored the initial dysbiosis [8,14,30].

As for microbial associations, we observed an expansion of *Enterococcus, Sedimentibacter*, and *Collinsella* species. Enterococci are pathobionts that have been linked to pyogenic infections in RA patients and patients with reactive arthritis. Moreover, a shift in the ratio of symbionts to pathobionts from the gut microbiota could generate an inflammatory imbalance owing to the fact that symbiotic bacteria usually trigger a Treg response, as opposed to a Th17 or Th1 response, whereas pathobionts can trigger Th17 or Th1 responses [31]. However, neither *Enterococcus* nor *Sedimentibacter* have been associated with dysbiosis in patients with RA or other autoimmune diseases. In contrast, the expansion of *C. aerofaciens,* which is thought to be involved in the pathogenesis of RA [14] and of psoriasis [32], was more noteworthy. This finding is in accordance with those of Chen et al. [14], who showed increased levels of this species in patients with established RA.

In our study, *C. aerofaciens* was associated with age, smoking, and high levels of ACPA, but not with the DMARDs used in RA. This suggests that DMARDs were not a confounding factor related to expansion of *C. aerofaciens*. An inverse association between *C. aerofaciens* and age would suggest that this species plays a more prominent role in younger patients, probably because they usually receive treatment earlier [33].

Smoking is one of the factors most strongly associated with peptide citrullination and the risk of RA [34]. This argument is reinforced by the association between *C. aerofaciens* and high levels of ACPA. Post-translational modifications of proteins by peptidyl-arginine-deiminases (PADs) can lead to the generation of autoantigens in the pathogenesis of RA [35]. Indeed, *Porphyromonas gingivalis* has been found in the biofilm of RA patients with gingivitis, and its presence is considered a risk factor for that disease, possibly because it expresses PADs [36,37]. By contrast, our RA patients showed a lower abundance of *Porphyromonas* than healthy subjects. This observation is in accordance with findings from studies that compared established RA with early RA [38] and may reflect differences related to treatment effects [39] or to disease progression itself.

The association between smoking, ACPA levels, and the abundance of *C. aerofaciens* in patients with lower levels of *P. gingivalis* led us to consider the possibility that *Collinsella* may be a key player in protein citrullination in the intestine and, therefore, a source of intestinal autoantigens that would facilitate ACPA production. This suspicion was reinforced by the finding of a higher content of genes related to arginine-deiminase activity in RA patients and the fact that a large percentage of them belonged to *Collinsella* species. Although this enzyme is widely expressed in anaerobic bacteria and produces energy by degrading arginine into citrulline [40,41], it is unclear whether a larger citrulline load in the intestine of RA patients could lead to more pronounced citrullination of proteins. Bennike et al. [42] identified 21 citrullinated peptides in the colonic tissues of both RA patients and controls that had previously been found in the lung tissue and synovial fluid of RA patients. The authors supported the hypothesis that colonic mucosa is potentially a site where immune tolerance to citrullinated proteins could be disrupted.

Other mechanisms by which *C. aerofaciens* might play a role in the pathogenesis of RA include molecular mimicry of HLA-DRB1*0401 [17], increases in intestinal permeability, alterations in neutrophil chemotaxis, and Interleukin 17 (IL-17) production [14]. Chen et al. [14] observed a strong correlation between the abundance of *Collinsella* and high levels of alpha-aminoadipic acid (a potential modulator of glucose homeostasis [43] and autoimmune marker), high asparagine levels (amino acid), and IL-17A production. An increase in the levels of beta-alanine, alpha-aminoadipic acid, and asparagine (a non-essential amino acid involved in blockade of apoptosis) [44] suggests that *Collinsella* significantly contributes to increased intestinal permeability.

Although none of these mechanisms were explicitly examined in our study, PICRUSt analysis demonstrated that, compared with controls, the patient’s microbiota facilitates copper transport and inhibits zinc transport. This finding may explain the higher copper levels and the lower zinc levels found in RA patients than in healthy subjects [45]. These trace elements are essential to many human biological processes, since they play a role in the functionality of enzymes and post-translational regulatory protein [46], with significant effects on cell regulation in both the adaptive and the innate immune systems. Thus, abnormal levels of these trace elements can have important consequences for the outcome of many inflammatory diseases, including RA [47].

Other significant findings resulting from the PICRUSt analysis concern amino acid metabolism and proteins involved in lipid biosynthesis, amino sugars, and nucleotide metabolism. These data are consistent with those of other studies [48,49]. Interestingly, the microbiota in our cohort more frequently harbored genes related to the proteins involved in lipid biosynthesis. Potential associations between gut dysbiosis, faulty lipid and glucose metabolism, and cardiovascular risk, particularly in patients with RA, is an under-explored area that should be specifically addressed in future studies.

This study is limited by its sample size, which may obscure a potential association between RA and dysbiosis. Moreover, for ethical reasons, all enrolled RA patients received immunomodulators, as it has been observed that these drugs can modify and restore microbiota. However, stratification by DMARDs revealed no significant differences, although differences may have arisen after stratification by sample size. Other authors have also reported on dysbiosis in untreated new-onset RA, and even in treated RA patients [5,6,7,8,9,10,11,12,13,14,15]. However, the strength of our study was that we analyzed patients with established RA who were prospectively followed with repeated measures of activity and damage-accrual data throughout the course of the illness and treated according to clinical practice guidelines. Nevertheless, as the analysis was cross-sectional in nature, more studies and larger cohorts are needed to confirm our findings and to establish a causal relationship between them. In future studies, it would be interesting to compare differences in the microbiota observed in our study with findings for other inflammatory joint diseases such as psoriatic arthritis.

In conclusion, our observations support the presence of dysbiosis in patients with RA. This dysbiosis is characterized by the expansion of lineages that are unusual and harmful, such as *Collinsella, Enterococcus*, and *Sedimentibacter*. In addition, we observed a decrease in lineages that are very common in healthy subjects and inducers of intestinal homeostasis (e.g., *Dorea* and *Sarcina*).

## Figures and Tables

**Table 1:** Baseline characteristics of the study population.

	Rheumatoid Arthritis (*n* = 40)	Healthy Controls (*n* = 40)	*p*
Clinical Characteristic			
Sex (Female), *n* (%)	30 (75.0)	30 (75.0)	1.000
Age (Years), Mean (SD)	58.5 (9.4)	58.5 (9.4)	0.998
Disease Duration, Months, Mean (SD)	78.9 (18.8)		
Smoking			0.018
Non-Smoker, *n* (%)	15 (37.5)	24 (60.0)	
Former Smoker, *n* (%)	16 (40.0)	5 (12.5)	
Smoker, *n* (%)	9 (22.5)	11 (27.5)	
BMI, Mean (SD)	29.7 (4.9)	28.1 (4.9)	0.104
Comorbidity			
Hypertension, *n* (%)	14 (35.0)	13 (32.5)	0.813
Dyslipidemia, *n* (%)	14 (35.0)	8 (20.0)	0.133
Type 2 Diabetes Mellitus, *n* (%)	1 (2.5)	0 (0.0)	0.314
Inflammatory Activity			
DAS28-ESR Average Value, Mean (SD)	3.6 (0.5)		-
DAS28-ESR at Index-Sate, Mean (SD)	3.0 (1.1)		-
HAQ Average Value, Mean (SD)	0.89 (0.6)		-
HAQ at Index-Date, Mean (SD)	1.06 (0.5)		-
Laboratory Characteristics			
RF-Positive, *n* (%)	32 (80.0)	2 (5.0)	<0.001
ACPA-Positive, *n* (%)	28 (70.0)	0 (0.0)	<0.001
CRP, mg/L, Mean (SD)	5.02 (4.5)	5.17 (7.0)	0.911
ESR mm/h, mean (SD)	17.1 (11.8)	12.2 (9.4)	0.041
Cholesterol, mg/dL, Mean (SD)	206.5 (39.7)	212.9 (37.0)	0.460
HDL, mg/dL	56.4 (14.5)	61.4 (16.5)	0.156
LDL, mg/dL	122.8 (30.5)	130.3 (29.6)	0.272
Triglycerides, mg/dL	137.1 (78.9)	104.3 (49.3)	0.030
Drugs			
Corticosteroids, *n* (%)	3 (7.5)		
Sulfasalazine, *n* (%)	6 (15.0)		
Leflunomide, *n* (%)	5 (12.5)		
Methotrexate, *n* (%)	29 (72.5)		
Hydroxychloroquine, *n* (%)	2 (5.0)		
Biologic Drugs, *n* (%)	15 (37.5)		

SD Standard deviation; BMI Body mass Index; ACPA Anti-cyclic citrullinated peptide antibody; CRP C-reactive protein; Disease Activity Score-28 with Erythrocyte Sedimentation Rate (DAS28-ESR); ESR Erythrocyte sedimentation rate; HAQ Health Assessment Questionnaire; HDL high-density lipoprotein; LDL, Low-density lipoprotein; RF Rheumatoid factor; Values are mean ± DESVEST. Significant differences vs. healthy donors (*p* < 0.05).

**Table 2:** Demographic and clinical characteristics of patients with RA harboring *Collinsella aerofaciens*.

Variables	*Collinsella aerofaciens (OTUs)*, Median (p25–p75)	*p*
Women Men	113.0 (44.0–345.5) 63.0 (27.5–190.0)	0.636
Smoking		0.036
Non-Smoker, *n* (%)	49.0 (19.0–154.0)	
Former Smoker, *n* (%)	204.0 (60.0–1020.0)	
Smoker, *n* (%)	109.5 (56.2–170.5)	
Bone Erosions Non-Bone Erosions	132.5 (48.0–346.2) 84.5 (18.0–144.2)	0.409
High ACPA (≥340) Low ACPA (<340)	154.0 (60.0–626.0) 55.0 (8.5–132.2)	0.024
High RF (≥60) Low RF (<60)	114.0 (38.0–267.7) 84.5 (36.0–261.7)	0.850
Double Seropositivity (RF+ ACPA+)	61.0 (28.5–202.0)	0.639
High DAS28 (≥3.2) Low DAS28 (<3.2)	114.0 (34.5–355.2) 86.0(43.5–225.2)	0.470
sDMARDs Non-sDMARDs	232.5 (60.0–900.0) 111.0 (31.5–206.7)	0.313
Methotrexate Non-Methotrexate	118.0 (36.0–206.7) 61.5 (39.0–373.7)	0.689
bDMARDs Non-bDMARDs	175.0 (60.0–930.5) 58.5 (31.5–161.5)	0.018

ACPA Anti-cyclic citrullinated peptide antibody; Disease Activity Score-28 with Erythrocyte Sedimentation Rate (DAS28-ESR); bDMARDs Biologic disease-modifying anti-rheumatic drugs; sDMARDs Synthetic disease-modifying anti-rheumatic drugs; OTUs Operational taxonomic units; RF Rheumatoid factor. Significant differences *p* < 0.05.

**Table 3:** Multiple linear regression analysis of clinical characteristics in relation to *Collinsella aerofaciens* OTUs (dependent variable) in RA patients.

Independent Variables	B	*p*	95% Confidence Intervals for B	
			Lower Boundary	Upper Boundary
Age	−0.347	0.018	−21.6	−2.1
Smoking	0.300	0.036	8.8	256.4
High ACPA	0.323	0.025	27.4	390.0

R^2^ Nagelkerke = 0.32, Predictor variables: gender, age, smoking, high ACPA, HAQ, bDMARDs. ACPA Anti-cyclic citrullinated peptide antibody; DAS28 Disease activity score 28; bDMARDs Biologic disease-modifying anti-rheumatic drugs; HAQ Health Assessment Questionnaire; OTUs Operational taxonomic units. Significant differences *p* < 0.005.
